# Loss of nuclear localization of TET2 in colorectal cancer

**DOI:** 10.1186/s13148-016-0176-7

**Published:** 2016-01-26

**Authors:** Yuji Huang, Guanghui Wang, Zhonglin Liang, Yili Yang, Long Cui, Chen-Ying Liu

**Affiliations:** Department of colorectal and anal surgery, Xinhua Hospital, School of Medicine, Shanghai Jiaotong University, Shanghai, China; Shanghai Colorectal Cancer Research Centre, Shanghai, China

**Keywords:** TET2, Colorectal cancer, 5hmC, DNA methylation, Nuclear localization

## Abstract

5-Hydroxymethylcytosine (5hmC) is lost in multiple human cancers, including colorectal cancer (CRC). Decreased ten-eleven translocation 1 (TET1) messenger RNA (mRNA), but not other two TET family members, has been observed in the colorectal cancer and is crucial for colorectal cancer initiation. Here, we show that nuclear localization of TET2 was lost in a significant portion of CRC tissues, in association with metastasis. In CRC cells, nuclear expression of TET2 were absent but not TET3. Nuclear export inhibitor can increase the 5hmC level in CRC cells, probably through regulating TET2. Our results indicate a new mechanism of TET2 dysregulation in colorectal cancer.

**Electronic supplementary material:**

The online version of this article (doi:10.1186/s13148-016-0176-7) contains supplementary material, which is available to authorized users.

5-Hydroxymethylcytosine (5hmC) is lost in multiple human cancers, including colorectal cancer. Inactivation of the ten-eleven translocation (TET) family members, the DNA hydroxylases catalyze 5mC into 5hmC, was closely related to the cancer initiation and progression. Recently, F Neri et al. reported the downregulation of TET1 messenger RNA (mRNA) expression level, but not other two TET family members, in colon cancer and showed that decreased TET1 mRNA was crucial for colon cancer initiation and TET1 functioned as a tumor suppressor by inhibiting the WNT pathway [1]. However, another group observed the decreased mRNA level of all TET family members in the colorectal cancer [2]. These contradictory findings could be due to the interfering by the stromal and infiltrating immune cells in the tumor tissues, the unstable mRNA in the tissue samples, and the RNA degradation during the RNA extraction. Besides, it was reported that the reduced levels of 5hmC in colorectal cancers is not correlated with TET mRNA levels [3], which indicates that dysregulation of TET protein could play a vital role in colorectal cancer. Thus, we analyzed both the mRNA and protein level of the TET family members in our colorectal cancer specimens.

Consistent with the finding of F Neri and another previous report [4], only TET1 was significantly decreased in the colorectal cancer tissues and cell lines at mRNA level (Additional file 1: Figure S1A and B). No detectable TET1 protein was observed in the CRC cell lines with low TET1 mRNA level (Additional file 1: Figure S1C), indicating that the decreased TET1 mRNA level resulted in low TET1 protein level in colorectal cancer. After testing several commercial TET2 antibodies, TET2 antibody (ab94580) was used for the immunohistochemical analysis of a CRC tissue array for the high sensitivity and specificity of this antibody (Additional file 1: Figure S1D). As shown in Fig. 1a, while normal and some CRC tissues contain positive nuclear staining of TET2, interestingly, a large portion of CRC tissues showed loss of nuclear staining of TET2 (Fig. 1b). After comparing TET2 nuclear expression with clinical data, we found that loss of nuclear TET2 expression was correlated with a more aggressive distal metastasis phenotype (*P* = 0.021) (Additional file 2: Table S1). No difference in the protein expression level and localization of TET3 between the normal and cancer tissues was observed (Additional file 1: Figure S1E). To confirm the finding of immunohistochemical (IHC) analysis, we detected the TET2 protein expression in the CRC cell lines. Consistent to IHC data, nuclear localization of TET2 was lost in 5 CRC cell lines, 1 lung cancer cell line, and 293T cells we tested (Fig. 1c). However, strong nuclear localization of TET3, another TET family member expressed in CRC cells, was detected in these cell lines. Also, we observed increased 5hmC level in multiple CRC cell lines after treatment with the nuclear export inhibitor, leptomycin B (LMB) (Fig. 1d). Knockdown TET2 further demonstrated that increasing 5hmC induced by LMB treatment was due to the TET2 protein (Fig. 1e).

**Fig. 1:**
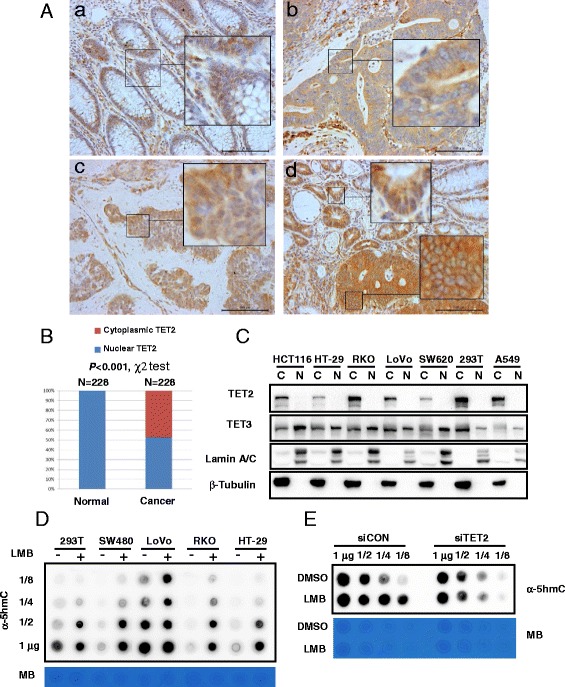
Loss of TET2 nuclear expression in colorectal cancer. **a** Immunohistochemical analysis of TET2 in colorectal cancer samples and normal mucosa tissues. Representative images of normal mucosa tissues (*a*), cytoplasmic expression of TET2 in CRC (*b*), nuclear expression of TET2 (*c*) in CRC, and TET2 expression in the invasive marginal region of CRC (*d*) were indicated. *Scale bar*: 100 μm. **b** Distribution of TET2 subcellular localization in normal mucosa tissues and colorectal cancer tissues. **c** Cytoplasmic (Cyto) and nuclear (Nuc) fractions of several cancer cells were separated for Western blot analysis as indicated. The following antibodies were used for Western blot: TET2 (ab94580), TET3 (GTX121452), Lamin A/C (CST #2032), and β-tubulin (Proteintech, 10068-1-AP). **d** Cells were treated with leptomycin B (LMB, 200 nM, 24 h), and then, DNA was extracted for 5hmC detection and methylene blue (*MB*) staining. **e** LoVo cells were transfected with siRNA targeting TET2 for 2 days and then treated with leptomycin B (200 nM, 24 h). DNA was extracted for 5hmC detection and methylene blue staining

DNA methylation is a therapeutic target for cancer treatment [5]. DNA hypermethylation occurs to the promoter of tumor suppressor genes, resulting in decreased expression of the tumor suppressor and cancer initiation and progression [5]. Inhibitors of DNA methyltransferases like 5-azacytidine and 5-aza-2′-deoxycytidine have been shown the efficacy in the treatment of multiple cancers to induce the expression of the tumor suppressor genes [6]. DNA hypermethylation of tumor suppressor genes could be due to the loss of active DNA demethylation which is consistent with global loss of 5hmC level in cancers [4, 7]. Thus, re-activation of the active DNA demethylation process could also lead to the re-expression of the tumor suppressor genes. The nuclear export inhibitors is also a promising cancer treatment drugs which have been shown to inhibit nuclear export of tumor suppressors like TP53 [8]. Here, we showed that LMB treatment can increase the global level of 5hmC probably through regulating TET2 in the CRC cell lines, which could promote the active demethylation and expression of tumor suppressor genes. Our results provided a novel mechanism that nuclear export inhibitor functioning through restoring the 5hmC level and potential expression of tumor suppressor genes which need to be further studied in the future.

Several mechanisms have been reported for the dysregulation of TET2 in cancers, including mutation of TET2 gene [9] and decreasing TET2 mRNA expression level [10, 11]. Nuclear exclusion of TET1 protein was found in the IDH1 wild-type gliomas [12]. Nuclear translocation of TET2 was first reported in the B cells [13]; furthermore, our results demonstrated that nuclear translocation of TET2 was also dysregulated in colorectal cancer, probably through the post-translational modification. Since TET2 needs to interact with DNA binding partners to regulate gene expression in the nucleus, loss of TET2 nuclear expression impaired TET2’s function. Thus, TET2 could also be a tumor suppressor in colorectal cancer, and the tumor suppressor function of both TET1 and TET2 was impaired in colorectal cancer through different mechanism.

## Additional files

Additional file 1: Figure S1. (A) The mRNA expression level of TET family members in 9 paired normal mucosa tissues and colorectal cancer tissues were detected by qPCR. (B) The mRNA expression level of TET family members in normal colon cell line (CCD-841) and six colorectal cancer cell lines were detected by qPCR. (C) The TET1 protein expression level in the colorectal cancer cell lines were detected by western blot. (D) Western blot analysis of several TET2 antibodies showed that the TET2 antibody (ab94580) used for IHC was specific for TET2 protein. FLAG-Tet2 was transfected into 293T cells by Lipofectamine 2000. 36h later, cells were lysed and detected by using FLAG antibody and TET2 antibody. (E) Immunohistochemical analysis of TET3 in colorectal cancer samples and normal mucosa tissues. Representative images of normal mucosa tissue and colorectal cancer tissue were shown. Scale bar: 100 μm. (PDF 419 kb)

Additional file 2: Table S1. Correlation of TET2 cytoplasmic expression with CRC patients’ pathological features. (DOC 84 kb)

Additional file 3: **Supplementary materials and methods.** (DOCX 22 kb)
