# Supplementary material for: Loss of nuclear localization of TET2 in colorectal cancer
Source: Clin Epigenetics. 2016 Jan 26;8:9. doi: 10.1186/s13148-016-0176-7 (PMC4727298; doi:10.1186/s13148-016-0176-7)
Supplement: Supplementary file 1 — (A) The mRNA expression level of TET family members in 9 paired normal mucosa tissues and colorectal cancer tissues were detected by qPCR. (B) The mRNA expression level of TET family members in normal colon cell line (CCD-841) and six colorectal cancer cell lines were detected by qPCR. (C) The TET1 protein expression level in the colorectal cancer cell lines were detected by western blot. (D) Western blot analysis of several TET2 antibodies showed that the TET2 antibody (ab94580) used for IHC was specific for TET2 protein. FLAG-Tet2 was transfected into 293T cells by Lipofectamine 2000. 36h later, cells were lysed and detected by using FLAG antibody and TET2 antibody. (E) Immunohistochemical analysis of TET3 in colorectal cancer samples and normal mucosa tissues. Representative images of normal mucosa tissue and colorectal cancer tissue were shown. Scale bar: 100 μm. (PDF 419 kb) [file 13148_2016_176_MOESM1_ESM.pdf]

Additional file 1: Figure S1

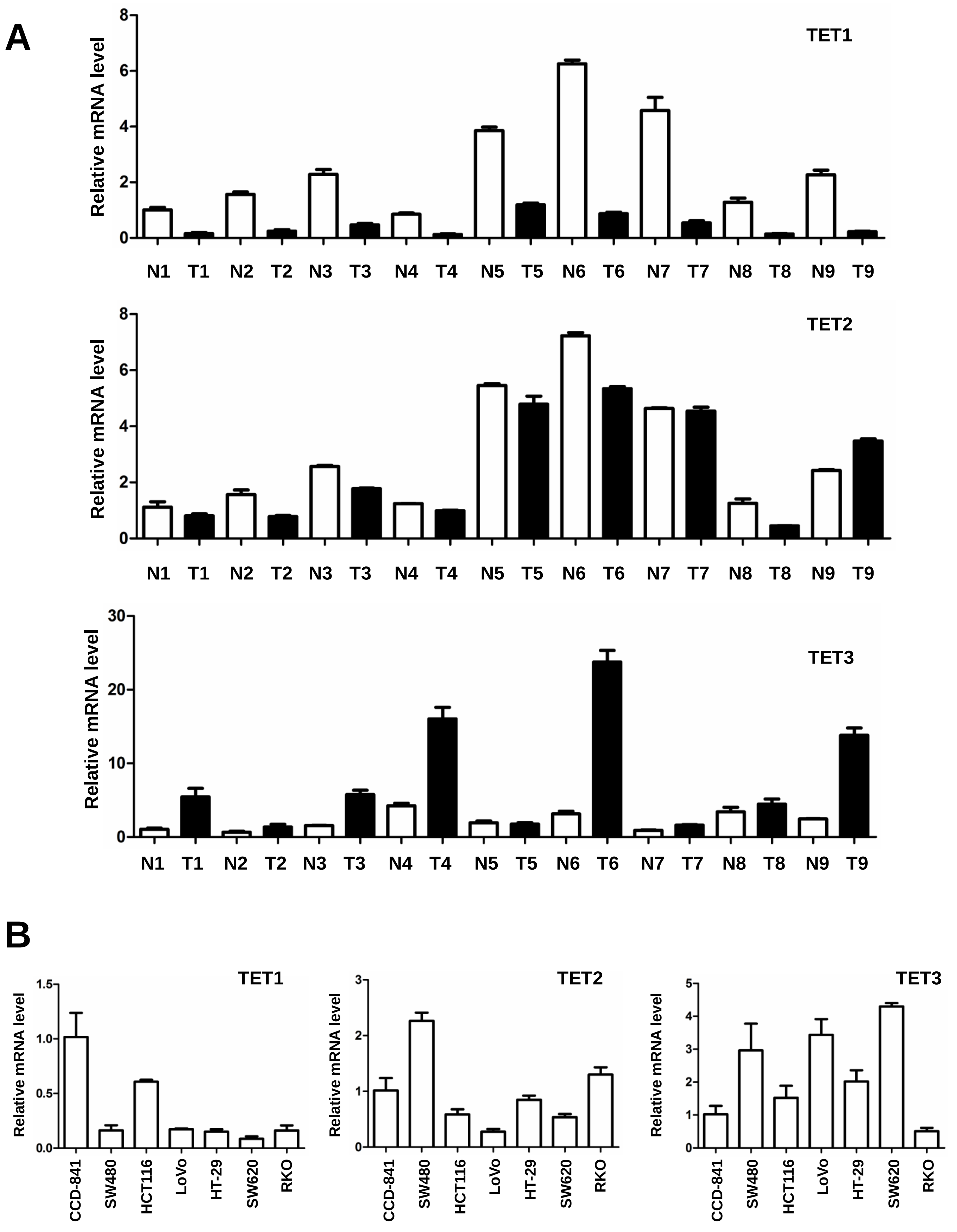

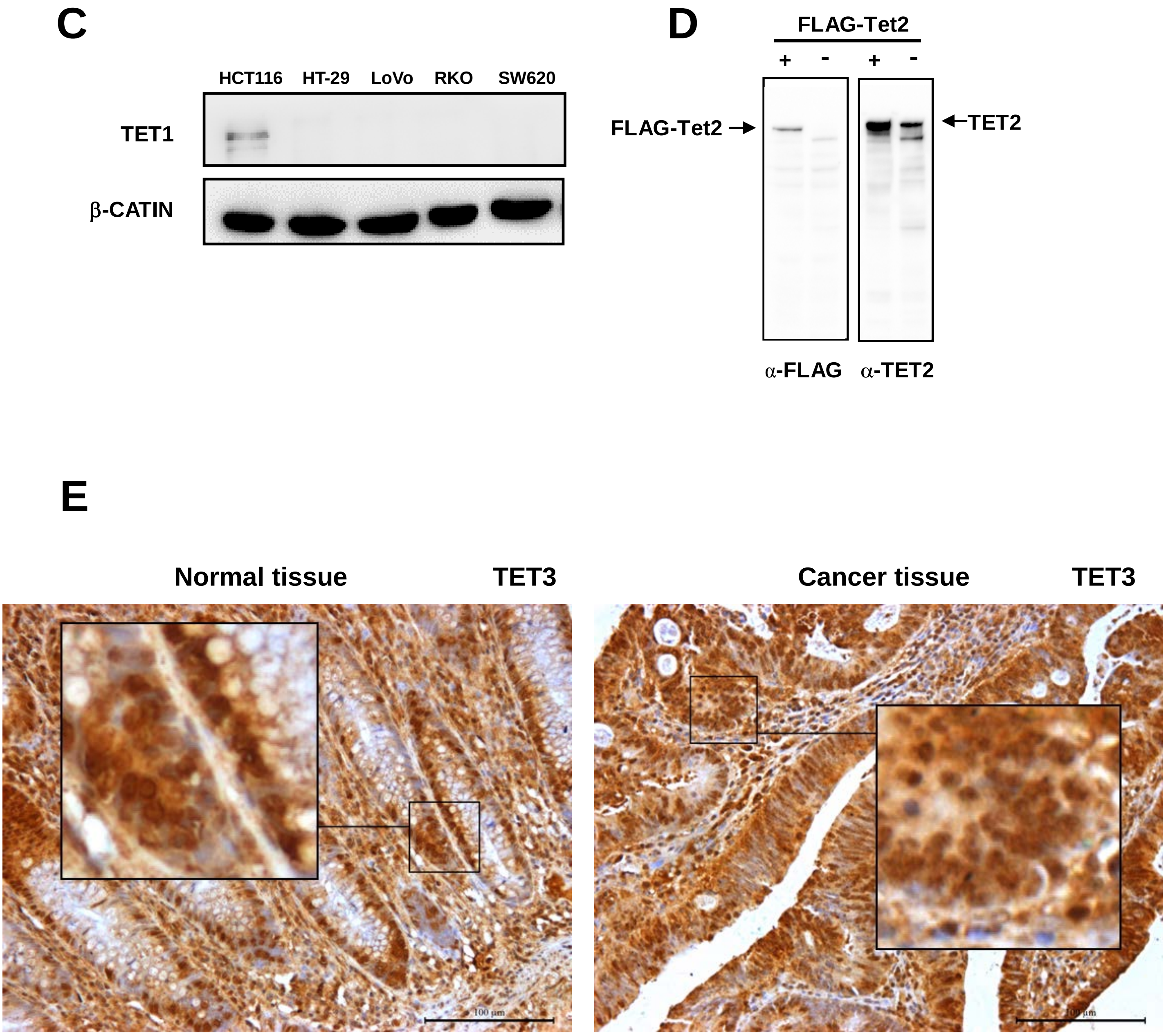
C
HCT116
HT-29
LoVo
RKO
SW620
TET1
β-CATIN
D
FLAG-Tet2
+
-
+
-
FLAG-Tet2
TET2
α-FLAG
α-TET2
E
Normal tissue
TET3
Cancer tissue
TET3

Supplementary figure legend

(A) The mRNA expression level of TET family members in 9 paired normal mucosa tissues and colorectal cancer tissues were detected by qPCR. (B) The mRNA expression level of TET family members in normal colon cell line (CCD-841) and six colorectal cancer cell lines were detected by qPCR. (C) The TET1 protein expression level in the colorectal cancer cell lines were detected by western blot. (D) Western blot analysis of several TET2 antibodies showed that the TET2 antibody (ab94580) used for IHC was specific for TET2 protein. FLAG-Tet2 was transfected into 293T cells by Lipofectamine 2000. 36h later, cells were lysed and detected by using FLAG antibody and TET2 antibody. (E) Immunohistochemical analysis of TET3 in colorectal cancer samples and normal mucosa tissues. Representative images of normal mucosa tissue and colorectal cancer tissue were shown. Scale bar: 100 μm.
