# Supplementary material for: Loss of nuclear localization of TET2 in colorectal cancer
Source: Clin Epigenetics. 2016 Jan 26;8:9. doi: 10.1186/s13148-016-0176-7 (PMC4727298; doi:10.1186/s13148-016-0176-7)
Supplement: Supplementary file 2 — Correlation of TET2 cytoplasmic expression with CRC patients’ pathological features. (DOC 84 kb) [file 13148_2016_176_MOESM2_ESM.doc]

| **Additional file 2: Table S1**  **Correlation of TET2 cytoplasmic expression with CRC patients’ pathological features** | | | | |
| --- | --- | --- | --- | --- |
| **Variables** | **TET2** | | | ***P* values** |
|  | **All cases**  (n=392) | **Cytoplasm**  (n=196) | **Nuclear**  (n=196) |  |
| Age（yr）^d^ | 392 |  |  | 0.154^a^ |
| ≤63 | 172 | 79(45.9%) | 93(54.1%) |  |
| >63 | 220 | 117(53.2%) | 103(46.8%) |  |
| Gender | 392 |  |  | 0.919^a^ |
| Male | 215 | 108(50.2%) | 107(49.8%) |  |
| Female | 177 | 88(49.7%) | 89(50.3%) |  |
| Tumor site^e^ | 392 |  |  | ***<0.001^b^** |
| Proximal colon | 68 | 48(70.6%) | 20(29.4%) |  |
| Distal colon | 141 | 56(39.7%) | 85(60.3%) |  |
| Rectum | 183 | 92(50.3%) | 91(49.7%) |  |
| Tumor size(cm) | 392 |  |  | 0.223^a^ |
| ≤5 cm | 216 | 102(47.2%) | 114(52.8%) |  |
| >5 cm | 176 | 94(53.4%) | 82(46.6%) |  |
| Differentiation | 392 |  |  | **^*^0.001^b^** |
| Well | 30 | 11(36.7%) | 19(63.3%) |  |
| Moderate | 323 | 175(54.2%) | 148(45.8%) |  |
| Poor | 39 | 10(25.6%) | 29(74.4%) |  |
| Tumor Type | 392 |  |  | **^*^0.001^a^** |
| Non-mucinous | 368 | 192(52.2%) | 176(47.8%) |  |
| Mucinous^f^ | 24 | 4(16.7%) | 20(83.3%) |  |
| TNM staging | 392 |  |  | 0.083^c^ |
| I | 53 | 22(41.5%) | 31(58.5%) |  |
| II | 136 | 69(50.7%) | 67(49.3%) |  |
| III | 147 | 69(46.9%) | 78(53.1%) |  |
| IV | 56 | 36(64.3%) | 20(35.7%) |  |
| Invasion | 392 |  |  | 0.413^c^ |
| T_1_ | 8 | 2(25.0%) | 6(75.0%) |  |
| T_2_ | 66 | 31(47.0%) | 35(53.0%) |  |
| T_3_ | 131 | 70(53.4%) | 61(46.6%) |  |
| T_4_ | 187 | 93(49.7%) | 94(50.3%) |  |
| No. of positive nodes | 392 |  |  | 0.400^c^ |
| 0 | 197 | 95(48.2%) | 102(51.8%) |  |
| 1-3 | 129 | 63(48.8%) | 66(51.2%) |  |
| >3 | 66 | 38(57.6%) | 28(42.4%) |  |
| Distal metastasis | 392 |  |  | ***0.021^a^** |
| M_0_ | 336 | 160(47.6%) | 176(52.4%) |  |
| M_1_ | 56 | 36(64.3%) | 20(35.7%) |  |
| Liver metastasis | 392 |  |  | **^*^0.022^a^** |
| No | 350 | 168(48.0%) | 182(52.0%) |  |
| Yes | 42 | 28(66.7%) | 14(33.3%) |  |
| Pelvic cavity metastasis | 392 |  |  | 0.083^a^ |
| No | 375 | 191(50.9%) | 184(49.1%) |  |
| Yes | 17 | 5(29.4%) | 12(70.6%) |  |

a. Mann-Whitney *U*-test, b. Kruskal-Wallis, c. Spearman.

d. Median age at operation

e. Proximal colon tumors are those arising in the cecum, ascending colon, hepatic flexure, or transverse colon; distal colon tumors are those arising in the splenic flexure, descending colon, or sigmoid colon; and rectal tumors are those arising in the rectosigmoid or rectum.

f. The mucinous type includes mucinous adenocarcinoma and signet ring cell carcinoma.

* Statistically significant
