# Supplementary material for: Loss of nuclear localization of TET2 in colorectal cancer
Source: Clin Epigenetics. 2016 Jan 26;8:9. doi: 10.1186/s13148-016-0176-7 (PMC4727298; doi:10.1186/s13148-016-0176-7)
Supplement: Supplementary file 3 — Supplementary materials and methods. (DOCX 22 kb) [file 13148_2016_176_MOESM3_ESM.docx]

**Supplementary Materials and Methods**

**Colorectal cancer specimen collection**

All the human colorectal cancer and paired normal mucosa samples for tissue arrays were collected in Department of Colorectal Surgery, XinHua Hospital, Shanghai Jiao Tong University School of Medicine, from January 2008 to December 2012. Institutional review board approval and informed consent were obtained for all the collections. 228 paired CRC and normal colon specimens were used to prepare tissue arrays and analyzed by immunohistochemistry. Another 164 CRC samples were used to prepare cancer specific tissue arrays for the immunohistochemical analysis of cytoplasmic TET2. Nine newly collected fresh CRC samples and paired normal mucosa tissues were used for real-time PCR analysis.

# Immunohistochemistry

The paraffin sections were deparaffinized, rehydrated, and treated according to standard protocol. After incubating with the anti-TET2 antibody (1:50; Abcam, ab94580) and anti-TET3 antibody (1:100; Genetex, GTX121452) overnight, sections were washed 3 times with PBS and incubated with HRP-conjugated secondary antibody (GK500710; Gene Company Ltd., Shanghai, China) for 30 min at room temperature. Following three rinses in PBS, staining was developed with 3,3'-diaminobenzidine (DAB) solution for 10 min. The sections were then counterstained with 0.1% hematoxylin and sealed with coverslips. Immunohistochemical staining was evaluated at x200 magnifications with a light microscope (Carl Zeiss, Göttingen, Germany).

**RNA isolation, quantitative real time-PCR，and microarray**

Total RNA was extracted using RNAiso Plus (TaKaRa) and reverse-transcribed into cDNA with PrimeScript® RT-PCR Kit (TaKaRa). Quantitative RT-PCR was performed by using SYBR® Premix Ex Taq™ (TaKaRa) and the ABI 7500 cycler. GAPDH was served as an internal control. The following primers were used for detecting the mRNA level of TET family members:

TET1-F: CCGAATCAAGCGGAAGAATA

TET1-R: ACTTCAGGTTGCACGGTCTC

TET2-F: AGCCCCATCACGTACAAAAC

TET2-R: TGTGGTGGCTGCTTCTGTAG

TET3-F: CAGCAGCCGAGAAGAAGAAG

TET3-R: GGACAATCCACCCTTCAGAG

GAPDH-F: GTCATCCAACGGGAATGCA

GAPDH-R: TGATCGGTTACCGTGATCAAAA

# Western blotting

# Cells were harvested and lysed with 1% NP-40 lysis buffer (50mM Tris-HCl, 150mM NaCl, 1% NP-40, 1 mM PMSF, 50 mM NaF, 1mM Na_3_VO_4_ and 1X protease inhibitor cocktails (Sigma, P8340) ). Proteins were separated in 10% SDS-PAGE gel and transferred onto nitrocellulose membrane. The membrane was blocked with 5% skim milk (BD), incubated with primary antibodies (1:1000) in 5% skim milk at 4℃ overnight, washed and incubated with HRP-conjugated secondary antibodies (Beyotime, 1:2000) in 5% skim milk for 1h at RT. The membrane was visualized by enhanced chemiluminescence reagents (Millipore). The following antibodies were used for western blot: TET1 (Millipore, 09-872), TET2 (ab94580), TET3 (GTX121452), Lamin A/C (CST #2032), β-Tubulin (Proteintech, 10068-1-AP), FLAG antibody (Sigma, F1804).

# Subcellular Fractionation

The cells were lysed with Harvest buffer (10 mM HEPES (pH 7.9), 50 mM NaCl, 0.5 M sucrose, 0.1 mM EDTA, 0.5% Triton X-100, 1 mM DTT, 1 mM PMSF, 50 mM NaF, 1mM Na_3_VO_4_ and 1X protease inhibitor cocktails (Sigma, P8340)). After incubation for 5 min on ice, the lysate was centrifuged at 1000 rpm for 10 min. The supernatant (cytoplasmic fraction) was transferred to a new tube and centrifuged at 14,000 rpm for another 15 min. Then, the supernatant was transferred to a new tube and boiled in 1× SDS loading buffer. The pellet (nuclear fraction) was washed three times with wash buffer (10 mM HEPES (pH 7.9), 10 mM KCl, 0.1 mM EDTA, and 0.1 mM EGTA), lysed with 1% NP-40 lysis buffer for 30 min and centrifuged at 14,000 rpm for15 min. The supernatant was transferred to a new tube and boiled in 1× SDS loading buffer.

**Cell culture and siRNA transfection**

Colorectal cancer cell lines HT-29, RKO, LoVo, SW480, SW620 and HCT116 were cultured in Dulbecco’s modified Eagle media (DMEM) supplemented with 10% fetal bovine serum and penicillin/streptomycin (100 unit/ml/100 µg/ml) at 37 ^0^C in a 5% CO^2^ atmosphere. Normal colon cell line CCD-841 was cultured in Eagle's Minimum Essential Medium supplemented with 10% fetal bovine serum and penicillin/streptomycin (100 unit/ml/100 µg/ml). TET2 siRNA transfections were performed using Lipofectamine-2000 following manufacturer’s instruction (Invitrogen). TET2 siRNA were purchased from Dharmacon. None-targeting control siRNA were purchased from Genepharma (Shanghai).

SMARTpool: ON-TARGETplus TET2 siRNA (L-013776-03-0005)

J-013776-22: ACAAGAAAGUAGAGGGUAU

J-013776-23: ACACCUAGUUUCAGAGAAU

J-013776-24: CCUCAGAAUAAUUGUGUGA

J-013776-25: CAGCAAAGGUACUUGAUAC

**Leptomycin B treatment and dot blot analysis**

Leptomycin B was purchased from Cell Signaling (9676, 200μM solution in ethanol). Cells were grown in the 10cm dishes at 70% confluence and treated with 200nM Leptomycin B or ethanol for 24 hours. Then, cells were collected for DNA extraction by using the TIANamp Genomic DNA Kit (DP304). The genomic DNA was concentrated by the vacuum centrifuge and adjusted to 1μg/μL. DNA was drop on the nitrocellulose membrane and crosslinked by UV for 30 min. 5hmC level was detected by using the 5hmC antibody (Active motif, 39769, 1:5000) as the western blot procedures described above. The equal loading amount of total DNA on the membrane was confirmed by staining with 0.02% methylene blue in 0.3M sodium acetate (pH 5.2).
